# Supplementary figures and images for: A Two-Step Transcriptome Analysis of the Human Heart Reveals Broad and Disease-Responsive Expression of Ectopic Olfactory Receptors
Source: Int J Mol Sci. 2023 Sep 5;24(18):13709. doi: 10.3390/ijms241813709 (PMC10530704; doi:10.3390/ijms241813709)

## Slide 1
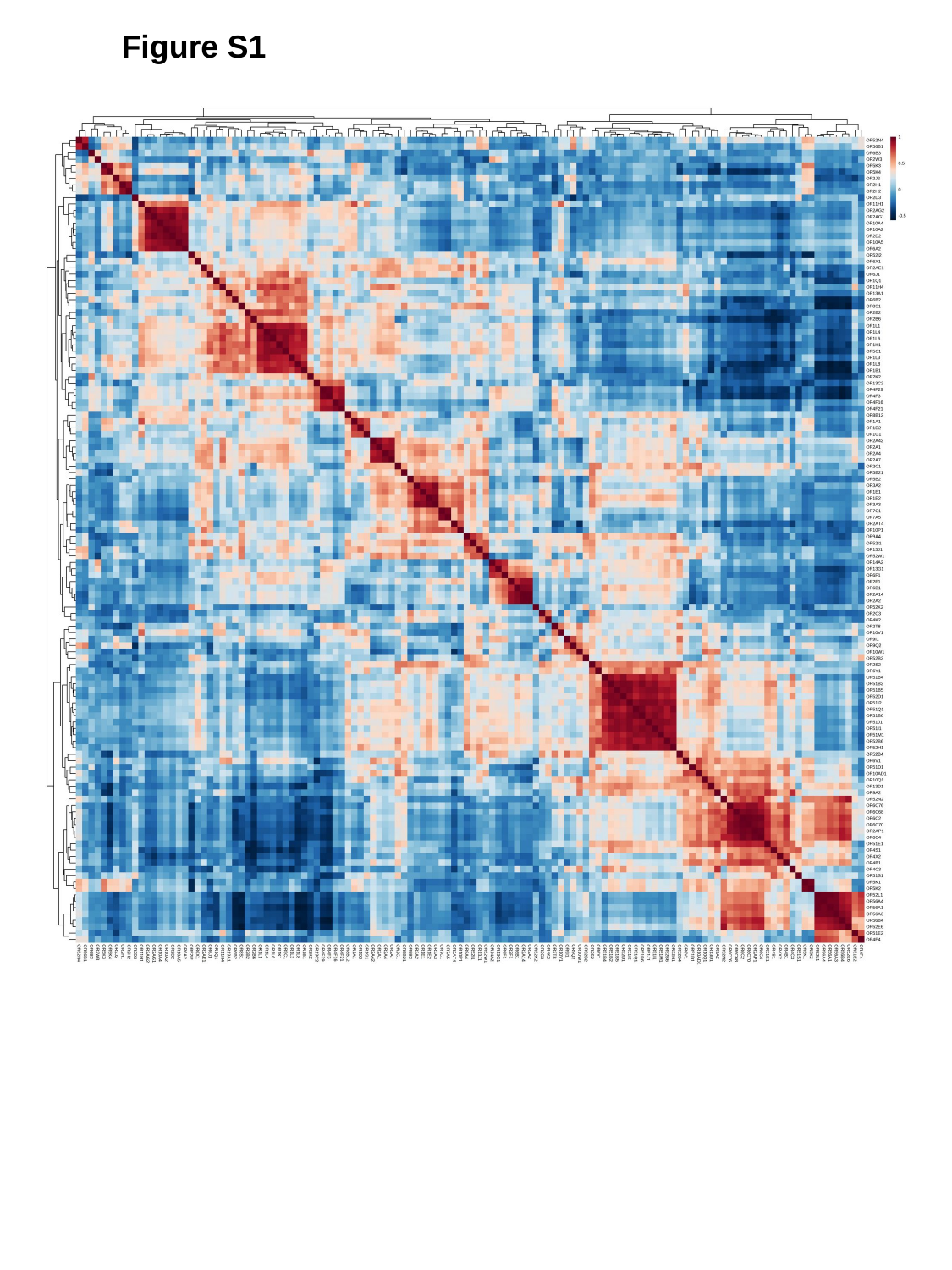

Figure S1

Supplement: Supplementary file 1 [file ijms-24-13709-s001.zip › Figure_S1-ORs_correlations_Matrix.pptx]
